# Identification of early biological changes in palmitate-treated isolated human islets

**DOI:** 10.1186/s12864-018-5008-z

**Published:** 2018-08-22

**Authors:** Ernest Sargsyan, Jing Cen, Kirsten Roomp, Reinhard Schneider, Peter Bergsten

**Affiliations:** 1 Department of Medical Cell Biology, Uppsala University, Box 571, 75123 Uppsala, Sweden; 2 Molecular Neuroscience Group, Institute of Molecular Biology, National Academy of Sciences, 0014 Yerevan, Armenia; 3 Luxembourg Centre for Systems Biomedicine, University of Luxembourg, Campus Belval, 7 avenue des Hauts fourneaux, 4362 Esch-Belval, Luxembourg City, Luxembourg

## Abstract

**Background:**

Long-term exposure to elevated levels of free fatty acids (FFAs) is deleterious for beta-cell function and may contribute to development of type 2 diabetes mellitus (T2DM). Whereas mechanisms of impaired glucose-stimulated insulin secretion (GSIS) in FFA-treated beta-cells have been intensively studied, biological events preceding the secretory failure, when GSIS is accentuated, are poorly investigated. To identify these early events, we performed genome-wide analysis of gene expression in isolated human islets exposed to fatty acid palmitate for different time periods.

**Results:**

Palmitate-treated human islets showed decline in beta-cell function starting from day two. Affymetrix Human Transcriptome Array 2.0 identified 903 differentially expressed genes (DEGs). Mapping of the genes onto pathways using KEGG pathway enrichment analysis predicted four islet biology-related pathways enriched prior but not after the decline of islet function and three pathways enriched both prior and after the decline of islet function. DEGs from these pathways were analyzed at the transcript level. The results propose that in palmitate-treated human islets, at early time points, protective events, including up-regulation of metallothioneins, tRNA synthetases and fatty acid-metabolising proteins, dominate over deleterious events, including inhibition of fatty acid detoxification enzymes, which contributes to the enhanced GSIS. After prolonged exposure of islets to palmitate, the protective events are outweighed by the deleterious events, which leads to impaired GSIS.

**Conclusions:**

The study identifies temporal order between different cellular events, which either promote or protect from beta-cell failure. The sequence of these events should be considered when developing strategies for prevention and treatment of the disease.

**Electronic supplementary material:**

The online version of this article (10.1186/s12864-018-5008-z) contains supplementary material, which is available to authorized users.

## Background

Elevated levels of circulating free fatty acids is one of the major factors implicated in alteration of insulin secretion in obese individuals [1]. In many of these individuals initial hyperinsulinemia is followed by later reduction of insulin secretion and development of type 2 diabetes mellitus (T2DM) [2, 3]. These clinical observations are supported by in vitro studies which show that a short-term exposure to fatty acids potentiates insulin secretion whereas a long-term exposure exerts deleterious effects [4–6].

Current treatment strategies aim to restore beta-cell function when functional decline is already evident. However, accumulating evidence indicate that therapeutic intervention at later stages when beta-cell function is already lost is inefficient and does not diminish the risk of T2DM [7]. Our observation that intracellular insulin content in fatty acid-treated isolated human islets is declined prior to the failure in insulin secretion suggests that deleterious mechanisms in fatty acid-exposed human islets are already activated at early time points when islets still hypersecrete insulin [4]. Therefore, it is desirable to identify such early mechanisms in order to prevent or reverse the disease by intervening at early stages when beta-cell function is not yet lost and intracellular changes have not passed a point of no return.

Mechanisms that are implicated in the long-term effects of fatty acids on beta cells have been intensively investigated. These mechanisms include alterations in mitochondrial function [8, 9], ER stress response [10], generation of ceramide species [11, 12], impairment of exocytotic machinery [13] and disturbances in GPR40 signalling [4, 14]. In contrast, events occurring prior to the failure of beta cells, when islets hypersecrete insulin, are much less understood.

**Fig. 1:**
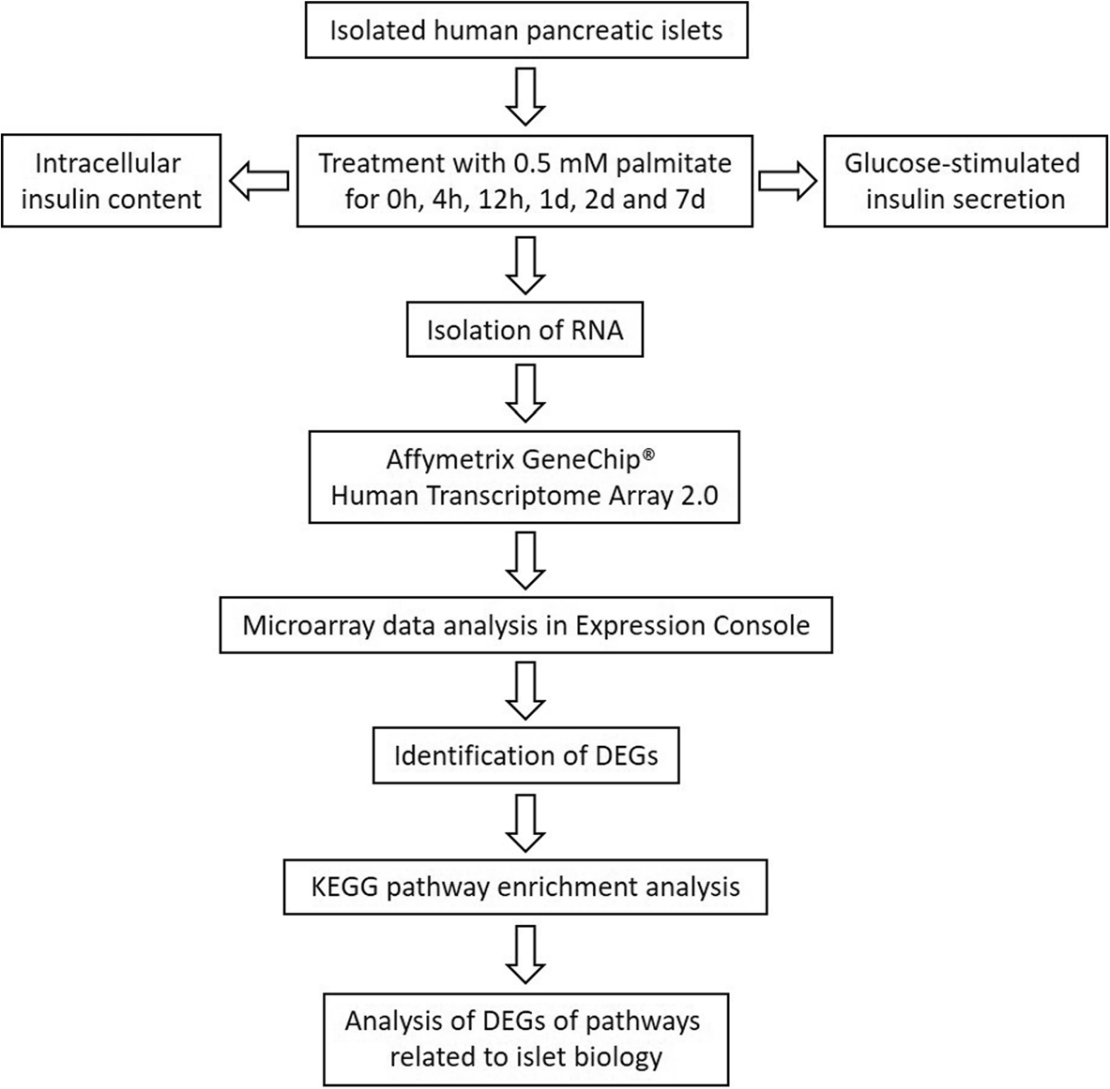
Workflow of the study

The aim of our study was to identify the biological events preceding the failure of beta cells in fatty acid-treated human islets. The aim was addressed by using genome-wide analysis of gene expression in isolated human islets exposed to fatty acid palmitate for various time periods (Fig. 1).

## Methods

### Culture of isolated human islets

Human islets were obtained from brain-dead otherwise healthy individuals from the Islet Prodo Lab Inc. (Irvine, CA, USA). The donors contributing islets to this study were two male donors aged 59 and 66 and three female donors aged 34, 39 and 48. Purity of islets varied between 85 and 90%. Islets were shipped in PIM (T) medium (Islet Prodo Lab Inc.) within 2–4 days after isolation. Upon arrival, islets were cultured in CMRL 1066 medium containing 5.5 mM glucose and supplemented with 10% FBS.

### Free fatty acid treatment of isolated human islets

Palmitate was prepared as 100 mM stock solution in 50% ethanol. The stock solution was diluted in culture medium to 0.5 mM concentration and complexed with 0.5 mM free fatty-acid free bovine serum albumin (BSA) for 30 min at 37 °C. Human islets were cultured in the absence and presence of palmitate for 4 and 12 h and 1, 2 and 7 days. Experiments were designed in a way that all treatments were terminated at day 7. Prior the treatments islets were cultured under control conditions and culture media were changed daily. After treatment, islets were picked up individually (to avoid non-islet structures) for glucose-stimulated insulin secretion, insulin content, and transcriptomics analyses.

### Insulin secretion and insulin content of human islets

After treatment, 15–20 human islets were picked individually and placed into a perifusion chamber. Islets were perifused at 37°C with a buffer (pH = 7.4) containing 125 mM NaCl, 5.9 mM KCl, 1.2  mM MgCl_2_, 1.3  mM CaCl_2_, 25 mM HEPES and 1% (*w*/*v*) fatty acid free BSA (fraction V; Boehringer Mannheim GmbH). The perifusion rate was 170 μl/min. During the first hour islets were perifused with a buffer containing 2 mM glucose, which was followed by a 20 min perifusion with a buffer containing 20 mM glucose. Perifusates were collected at − 15, − 10, − 5, 0, 2, 4, 6, 10, 15, 20 min to measure the amounts of secreted insulin. At 0 min the glucose concentration was raised from 2 to 20 mM. After perifusion, islets were washed with Dulbecco’s phosphate buffered saline (DPBS) and lysed in DPBS buffer containing 1% Triton X100 and 0.4% protease inhibitor cocktail (both obtained from Sigma Aldrich). Lysates were used for measurements of insulin and protein content. Insulin was determined by a competitive ELISA, as previously described [15]. For each perifusion, insulin secretory rate at stimulatory (20 mM) glucose was normalized to total protein. Insulin secretion was expressed as a ratio between insulin secretory rate at stimulatory glucose from treated islets and insulin secretory rate at stimulatory glucose from untreated islets for each donor. Insulin content was normalized to islet protein and expressed as fold control.

### Preparation of mRNA

After treatment, 50 human islets were individually collected and washed with PBS tree times. mRNA was isolated using mRNA isolation kit from Macherey-Nagel (Duren, Germany) according to the manufacturer’s instructions. RNA concentration was measured with ND-1000 spectrophotometer (NanoDrop Technologies, Wilmington, DE) and RNA quality was evaluated using the Agilent 2100 Bioanalyzer system (Agilent Technologies Inc., Palo Alto, CA).

### Microarray expression analysis

Total RNA, 100 nanograms from each sample, was used to generate amplified and biotinylated sense-strand cDNA from the entire expressed genome according to the GeneChip® WT PLUS Reagent Kit User Manual (P/N 703174 Rev. 1 Affymetrix Inc., Santa Clara, CA). GeneChip® ST Arrays (GeneChip® Human Transcriptome Array (HTA) 2.0) were hybridized for 16 h in a 45 °C incubator, rotated at 60 rpm. According to the GeneChip® Expression Wash, Stain and Scan Manual (PN 702731 Rev. 3, Affymetrix Inc., Santa Clara, CA) the arrays were then washed and stained using the Fluidics Station 450 and finally scanned using the GeneChip® Scanner 3000 7G. The HTA array covers almost 68,000 genes of which 27,000 are annotated.

### Microarray data analysis

The raw data was normalized in Expression Console, provided by Affymetrix (http://www.affymetrix.com), using the robust multi-array average (RMA) method as previously described [16, 17]. Genes with a more than 1.3-fold change after palmitate exposure compared to untreated islets were defined as differentially expressed (DEGs).

### KEGG pathway enrichment analysis

Differentially expressed genes were selected for bioinformatics analysis. KEGG (Kyoto Encyclopedia of Genes and Genomes) pathway over-representation analysis was done using ConsensusPathDB (http://consensuspathdb.org/) [18]. In the further analysis, DEGs in the pathways of interest were manually annotated using literature survey and UniProt database.

### Statistical analysis

Results of glucose-stimulated insulin secretion (GSIS) and insulin content are presented as means ± SEM. Gene’s expression is presented as mean ± SD. Due to high variability between islets and limited resources to increase the sample size, we increased the validity of the results by normalizing each experiment to its own control. Statistical significance was evaluated by using one-way *ANOVA with Dunnett’s multiple comparison* test. *p* < 0.05 was considered statistically significant.

KEGG pathway over-representation analysis in ConsensusPathDB was carried out using input gene lists that were compared to functional modules derived from KEGG pathway definitions. A *p*-value cut-off of < 0.01 and a minimum overlap with the input list of two genes were used. The calculated *p*-value reflects the significance of the observed overlap between an input gene list and a module’s members, as compared to random expectations. Therefore, small *p*-values indicate that of the genes in the input list, more are present in a module (pathway) than would be expected by chance alone and this may indicate dysregulated pathways [19].

## Results

### GSIS and insulin content of human islets exposed to palmitate

**Fig. 2:**
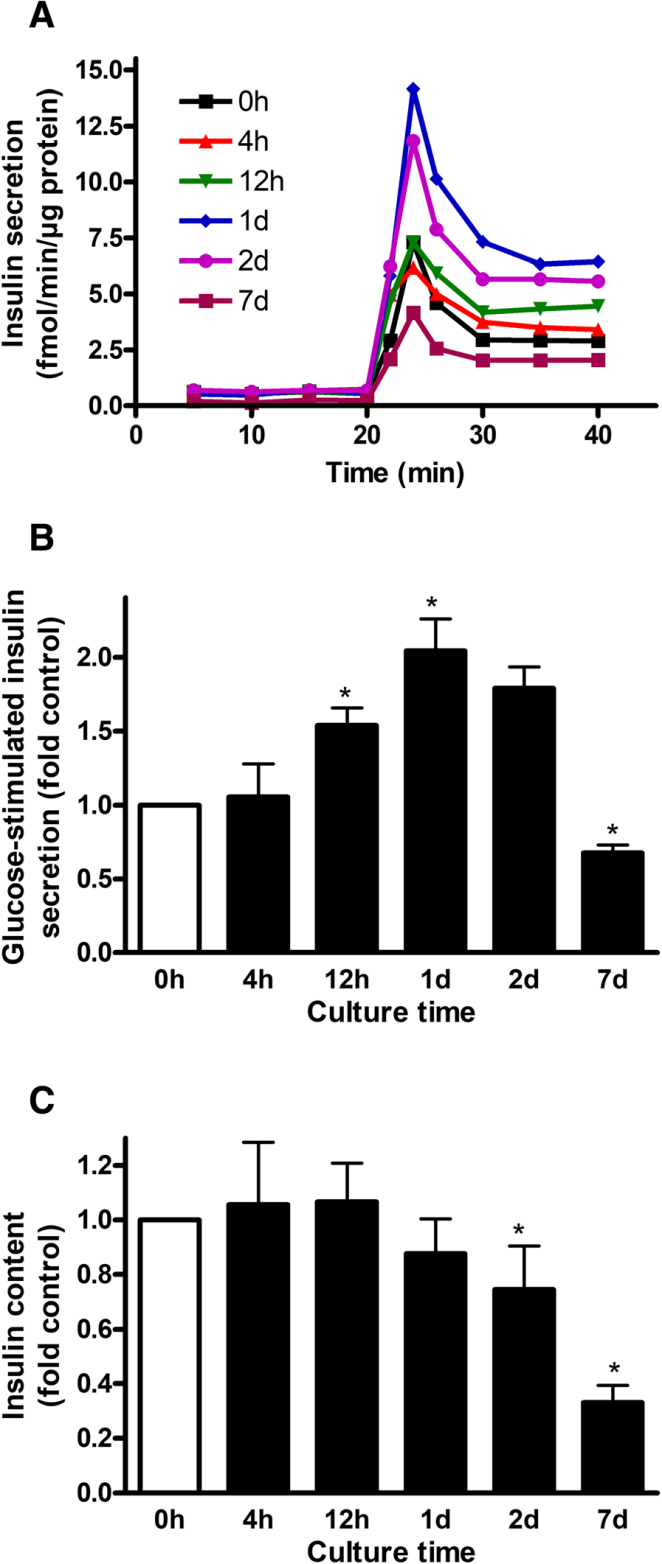
GSIS and intracellular insulin content in isolated human islets exposed to palmitate for 0, 4 and 12 h and 1, 2 and 7 days. After culture, islets were perifused with 2 mM glucose followed by 20 min perifusion with 20 mM glucose. Panel **a**. Representative graphs of dynamic insulin secretion from one donor. Panel **b**. Insulin secretory rate at stimulatory (20 mM) glucose was normalized to total protein. Insulin secretion was expressed as a ratio between insulin secretory rate at stimulatory glucose from treated islets and insulin secretory rate at stimulatory glucose from untreated islets for each donor. Panel **c**. Islets were subsequently lysed and insulin content measured. Insulin content after treatments was normalized to total protein content and expressed as fold insulin content in untreated islets. Results are means of 5 donors ± SEM. *p < 0.05 vs control

GSIS and insulin content was determined in isolated human islets exposed to palmitate for 0, 4 and 12 h and 1, 2 and 7 days. After 4 h, GSIS was not changed compared with control islets. After a longer exposure period, GSIS gradually increased reaching the maximal level after 1 day and then decreased to 70% of control level after 7 days (Figs. 2a, b). Intracellular insulin content was not changed up to 1-day culture with palmitate but gradually decreased to approximately 75 and 30% of control level after 2 and 7 days, respectively (Fig. 2c).

### Transcriptome of human islets exposed to palmitate

**Fig. 3:**
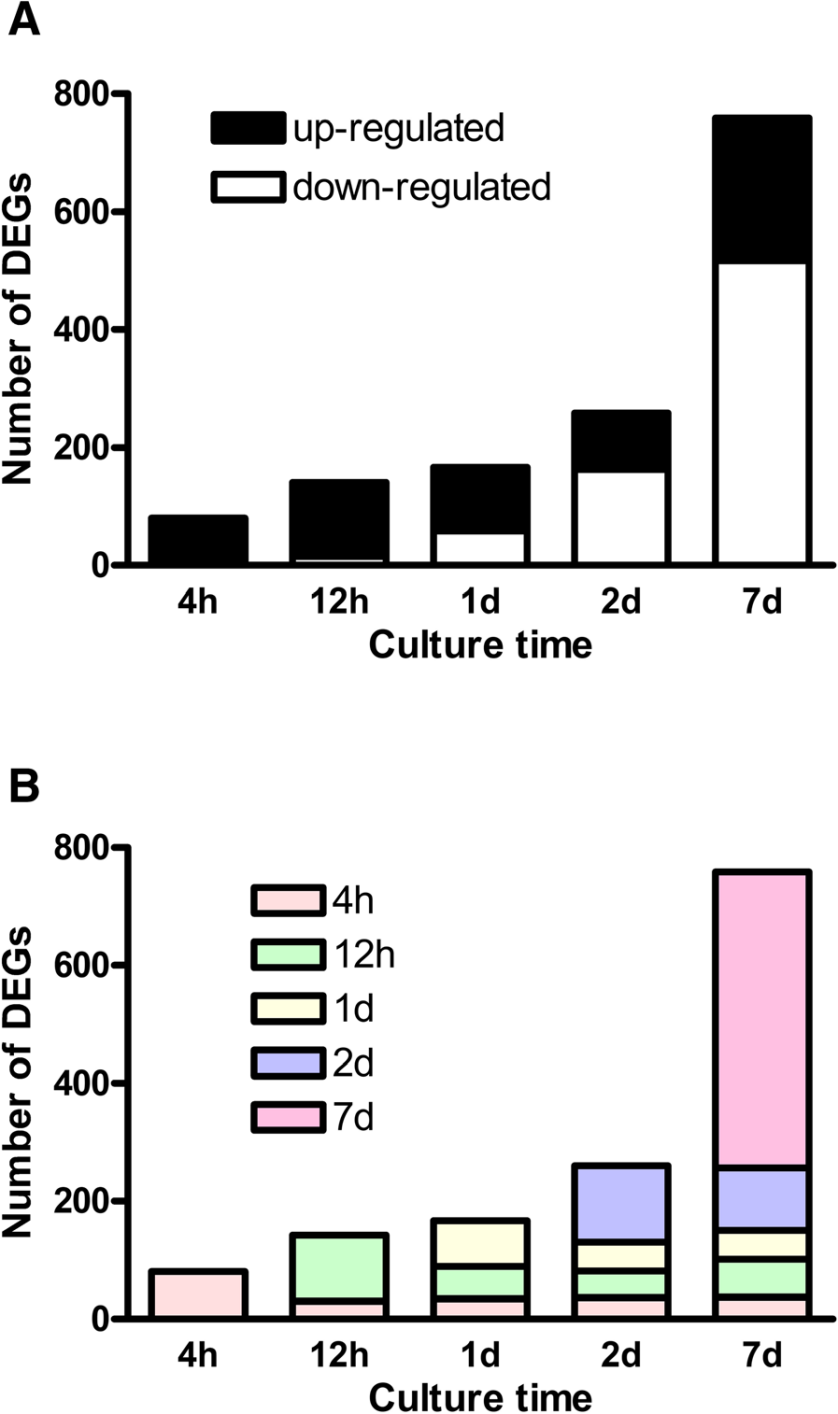
Transcriptome of isolated human islets exposed to palmitate for 0, 4 and 12 h and 1, 2 and 7 days. Transcriptomics analysis was performed by using GeneChip® Human Transcriptome Array 2.0 from Affymetrix. Genes were defined as differentially expressed if they changed more than 1.3-fold after palmitate treatment with the significance level of *p* ≤ 0.05. Panel **a**. Number of up- and down-regulated genes. Panel **b**. Each color reflects DEGs that appear for the first time at a certain culture duration. Results are from 5 donors

To delineate mechanisms for these time-dependent changes in GSIS and insulin content we performed a transcriptomics analysis of human islets exposed to palmitate for 0, 4 and 12 h and 1, 2 and 7 days. The genes were defined as differentially expressed if they were changed more than 1.3-fold after palmitate treatment. Using this cut-off we found that out of 27,000 transcripts 903 unique genes were differentially expressed at least in one of the culture periods. The number of DEGs increased with the exposure time (Fig. 3a). It was 80 after 4 h, 142 after 12 h, 167 after 1 day, 259 after 2 days and 759 after 7 days.

Further analysis of the DEGs demonstrated that the percentages of up- and down-regulated genes also changed with the exposure time (Fig. 3a). Whereas the proportion of down-regulated genes was only 10% after 4 and 12 h (8 genes and 15 genes, respectively), the percentage rose to 35% (58 genes) after 1 day, 63% (163 genes) after 2 days and 68% (517 genes) after 7 days.

Next, we followed DEGs over time (Fig. 3b). We found that ~ 300 genes were differentially expressed prior to the decline of beta-cell function, up to 1-day of palmitate exposure. Of those, 36 DEGs (40%) after 4 h, 64 DEGs (55%) after 12 h, and 49 DEGs (60%) after 1 day were differentially expressed also after 7 days. Interestingly, only 4 genes, all encoding members of aldo-keto reductase family 1 (B10, B15, C1 and C2), were changed in opposite directions over time. These genes were up-regulated after 12 h but down-regulated after 7 days of palmitate exposure i.e. in a similar way that was observed for GSIS.

### KEGG pathway enrichment analysis of differentially expressed genes

**Fig. 4:**
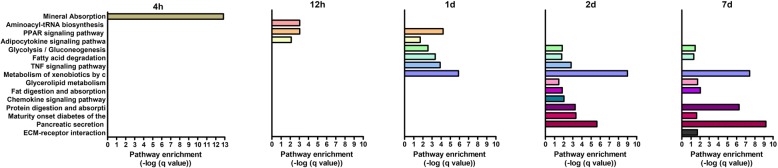
Enriched pathways in isolated human islets exposed to palmitate for 4 and 12 h and 1, 2 and 7 days. KEGG pathway enrichment analysis was performed by using DEGs. Of 55 predicted pathways, the dynamic changes of 15 pathways related to islet biology are demonstrated

Next, we mapped all the DEGs onto pathways using KEGG pathway enrichment analysis to obtain an overview of biological events in human islets during fatty acid exposure. The analysis predicted 56 pathways significantly enriched at least in one culture duration (Additional file 1: Table S1). Pathways enriched at each culture time points are listed separately (Additional file 2: Tables S2, Additional file 3: Table S3, Additional file 4: Table S4, Additional file 5: Table S5, Additional file 6: Table S6). Among these pathways we selected 15 related to islet biology (Fig. 4). To understand the early biological events during palmitate exposure, we have focused on those pathways which were enriched prior to the decline of islet function i.e. at 4 and 12 h and 1 day of palmitate exposure. Eight pathways were enriched already at these early time points. Of those, the four pathways, “Mineral absorption”, “Aminoacyl-tRNA biosynthesis”, “PPAR signaling pathway” and “Adipocytokine signaling pathway”, were enriched only prior to the decline of islet function. The three pathways, “Metabolism of xenobiotics by P450”, “Fatty acid degradation” and “Glycolysis/gluconeogenesis”, were enriched both before and after the decline of islet function and the pathway “TNF signalling pathway” was enriched after 1 and 2 days of exposure to palmitate but not at other time points. The remaining seven pathways were enriched only after 2 and/or 7 days of palmitate exposure. Expression levels of the DEGs were further detailed at the different culture time points for the eight selected pathways (Table 1).

**Table 1:** Expression profile of genes from the enriched pathways over time (fold untreated ± S.D., *n* = 5)

Pathway name	Gene symbol	Gene name	**4 h**	12 h	1d	2d	7d
Mineral absorption	MT1F	metallothionein 1F	2.18 ± 0.56*	1.27 ± 0.32	1.36 ± 0.42	1.4 ± 0.33	1.41 ± 0.58
	MT2A	metallothionein 2A	2.19 ± 0.37*	1.08 ± 0.31	1.23 ± 0.42	1.32 ± 0.48	1.25 ± 0.49
	MT1L	metallothionein 1 L (gene/pseudogene)	2.2 ± 0.45*	1.21 ± 0.29	1.26 ± 0.23	1.25 ± 0.29	1.17 ± 0.26
	MT1A	metallothionein 1A	2.34 ± 0.52*	1.11 ± 0.27	1.21 ± 0.2	1.23 ± 0.33	1.2 ± 0.38
	MT1E	metallothionein 1E	2.58 ± 0.65*	1.19 ± 0.22	1.22 ± 0.21	1.34 ± 0.34	1.3 ± 0.27
	MT1M	metallothionein 1 M	2.76 ± 0.63*	1.19 ± 0.29	1.25 ± 0.19	1.24 ± 0.27	1.18 ± 0.31
	MT1B	metallothionein 1B	1.9 ± 0.22*	1.08 ± 0.17	1.11 ± 0.11	1.16 ± 0.2	1.11 ± 0.19
	MT1X	metallothionein 1X	3.49 ± 1.76*	1.24 ± 0.43	1.33 ± 0.4	1.35 ± 0.42	1.28 ± 0.49
	MT1H	metallothionein 1H	2.89 ± 1.3*	1.27 ± 0.34	1.32 ± 0.37	1.3 ± 0.31	1.14 ± 0.44
	MT1G	metallothionein 1G	3.28 ± 1.44*	1.43 ± 0.4	1.48 ± 0.53	1.35 ± 0.4	1.12 ± 0.41
	SLC30A1	solute carrier family 30 (zinc transporter). member 1	1.98 ± 0.44*	1.26 ± 0.21	1.11 ± 0.12	1.24 ± 0.29	1.24 ± 0.25
Aminoacyl-tRNA biosynthesis	IARS	isoleucyl-tRNA synthetase	1.15 ± 0.18	1.58 ± 0.28*	1.25 ± 0.29	1.14 ± 0.17	1.22 ± 0.17
	AARS	alanyl-tRNA synthetase	1.1 ± 0.13	1.41 ± 0.21*	1.19 ± 0.23	1.04 ± 0.14	1.17 ± 0.11
	YARS	tyrosyl-tRNA synthetase	1.24 ± 0.15	1.49 ± 0.32*	1.28 ± 0.24	1.15 ± 0.11	1.21 ± 0.13
	MARS	methionyl-tRNA synthetase	1.17 ± 0.17	1.62 ± 0.33*	1.25 ± 0.24	1.12 ± 0.14	1.19 ± 0.16
	EPRS	glutamyl-prolyl-tRNA synthetase	1.09 ± 0.13	1.37 ± 0.12*	1.12 ± 0.2	1.09 ± 0.19	1.16 ± 0.11
	GARS	glycyl-tRNA synthetase	1.3 ± 0.18*	1.56 ± 0.37*	1.38 ± 0.22*	1.22 ± 0.17	1.3 ± 0.16
PPAR signaling	CPT1A	carnitine palmitoyltransferase 1A	1.25 ± 0.1*	1.4 ± 0.09*	1.32 ± 0.18*	1.21 ± 0.08	1.2 ± 0.12
	SCD	stearoyl-CoA desaturase (delta-9-desaturase)	1.03 ± 0.09	1.46 ± 0.16*	1.56 ± 0.22*	1.6 ± 0.21*	2 ± 0.33*
	ACSL1	acyl-CoA synthetase long-chain family member 1	1.3 ± 0.1*	1.47 ± 0.16*	1.51 ± 0.15*	1.44 ± 0.11*	1.2 ± 0.19
	ANGPTL4	angiopoietin-like 4	1.4 ± 0.19*	1.42 ± 0.19*	1.47 ± 0.33*	1.53 ± 0.31*	1.37 ± 0.26
	ME1	malic enzyme 1, NADP(+)-dependent, cytosolic	1.15 ± 0.13	1.52 ± 0.25*	1.47 ± 0.31*	1.26 ± 0.27	1.52 ± 0.32*
	SLC27A2	solute carrier family 27 (fatty acid transporter), member 2	1.25 ± 0.14	1.36 ± 0.21*	**1.27 ± 0.2**	1.23 ± 0.16	1.61 ± 0.4*
	GK	glycerol kinase	1.04 ± 0.09	1.28 ± 0.13*	1.34 ± 0.24*	0.98 ± 0.09	0.95 ± 0.13
Adipocytokine signaling	CPT1A	carnitine palmitoyltransferase 1A	1.25 ± 0.1*	1.4 ± 0.09*	1.32 ± 0.18*	1.21 ± 0.08	1.2 ± 0.12
	ACSL1	acyl-CoA synthetase long-chain family member 1	1.3 ± 0.1*	1.47 ± 0.16*	1.51 ± 0.15*	1.44 ± 0.11*	1.2 ± 0.19
	NFKBIA	nuclear factor of kappa light polypeptide gene enhancer in B-cells inhibitor, alpha	1.06 ± 0.07	1.33 ± 0.03*	1.27 ± 0.05*	1.39 ± 0.22*	1.64 ± 0.5
	IRS2	insulin receptor substrate 2	1.54 ± 0.24*	1.49 ± 0.25*	1.45 ± 0.26*	1.48 ± 0.2*	1.58 ± 0.32*
	G6PC2	glucose-6-phosphatase, Catalytic, 2	1.27 ± 0.06*	1.32 ± 0.14*	**1.22 ± 0.2**	1.12 ± 0.13	1.27 ± 0.12
TNF signalling	CXCL1	chemokine (C-X-C motif) ligand 1 (melanoma growth stimulating activity, alpha)	0.87 ± 0.11	1.39 ± 0.15*	1.58 ± 0.19*	1.77 ± 0.45*	1.43 ± 0.29
	PTGS2	prostaglandin-endoperoxide synthase 2 (prostaglandin G/H synthase and cyclooxygenase)	3.19 ± 1.24*	2.1 ± 0.36*	2.78 ± 0.8*	2.72 ± 0.26*	4.52 ± 1.7
	LIF	leukemia inhibitory factor	1.05 ± 0.08	1.21 ± 0.12	1.3 ± 0.1*	1.4 ± 0.24*	1 ± 0.04
	CXCL2	chemokine (C-X-C motif) ligand 2	0.97 ± 0.11	1.3 ± 0.16	1.34 ± 0.18*	**1.5 ± 0.34**	1.48 ± 0.51
	BIRC3	baculoviral IAP repeat containing 3	0.93 ± 0.1	1.18 ± 0.2	1.3 ± 0.19*	**1.11 ± 0.33**	0.82 ± 0.3
	ICAM1	intercellular adhesion molecule 1	1. 03 ± 0.04	1.24 ± 0.16	1.43 ± 0.11*	1.64 ± 0.32*	1.7 ± 0.7
	CX3CL1	chemokine (C-X3-C motif) ligand 1	1.13 ± 0.12	1.23 ± 0.18	1.38 ± 0.23*	**1.59 ± 0.68**	1.33 ± 0.67
	CCL20	chemokine (C-C motif) ligand 20	0.76 ± 0.3	1.57 ± 0.63	2.5 ± 0.9*	1.61 ± 0.32*	1.28 ± 0.4
Metabolism of xenobiotics by P450	CYP2C9	cytochrome P450, family 2, subfamily C, polypeptide 9	0.87 ± 0.05	0.86 ± 0.1	**0.8 ± 0.06**	0.73 ± 0.11*	0.62 ± 0.12*
	CYP1A1	cytochrome P450, family 1, subfamily A, polypeptide 1	1.23 ± 0.43	1 ± 0.34	0.7 ± 0.13*	0.6 ± 0.24*	0.35 ± 0.09*
	CYP1B1	cytochrome P450, family 1, subfamily B, polypeptide 1	1.14 ± 0.22	1.13 ± 0.23	**0.87 ± 0.16**	**0.89 ± 0.22**	0.64 ± 0.17*
	UGT2B7	UDP glucuronosyltransferase 2 family, polypeptide B7	0.91 ± 0.15	0.86 ± 0.11	**0.79 ± 0.06**	0.77 ± 0.1*	0.64 ± 0.06*
	UGT2A3	UDP glucuronosyltransferase 2 family, polypeptide A3	0.95 ± 0.2	0.96 ± 0.22	0.71 ± 0.18*	0.59 ± 0.13*	0.29 ± 0.06*
	GSTA1	glutathione S-transferase alpha 1	1.1 ± 0.2	1.11 ± 0.23	**0.98 ± 0.2**	0.73 ± 0.16*	0.45 ± 0.08*
	GSTA2	glutathione S-transferase alpha 2	1.1 ± 0.24	1.16 ± 0.31	**0.97 ± 0.23**	0.68 ± 0.2*	0.36 ± 0.06*
	UGT2B15	UDP glucuronosyltransferase 2 family, polypeptide B15	0.89 ± 0.22	0.89 ± 0.27	0.53 ± 0.12*	0.37 ± 0.13*	0.16 ± 0.03*
	UGT2B17	UDP glucuronosyltransferase 2 family, polypeptide B17	0.87 ± 0.23	0.88 ± 0.25	0.58 ± 0.11*	0.43 ± 0.15*	0.23 ± 0.06*
	UGT2B10	UDP glucuronosyltransferase 2 family, polypeptide B10	0.97 ± 0.07	0.98 ± 0.11	**0.96 ± 0.1**	**0.94 ± 0.09**	0.77 ± 0.09*
	ADH1B	alcohol dehydrogenase 1B (class I), beta polypeptide	0.86 ± 0.1	0.82 ± 0.1	0.74 ± 0.11*	0.69 ± 0.13*	0.6 ± 0.11*
	ADH1A	alcohol dehydrogenase 1A (class I), alpha polypeptide	0.86 ± 0.07	0.87 ± 0.1	0.73 ± 0.15*	0.67 ± 0.16*	0.58 ± 0.17*
	ADH1C	alcohol dehydrogenase 1C (class I), gamma polypeptide	0.76 ± 0.13*	0.65 ± 0.17*	0.5 ± 0.18*	0.39 ± 0.14*	0.17 ± 0.08*
	CYP3A4	cytochrome P450, family 3, subfamily A, polypeptide 4	0.83 ± 0.11	1 ± 0.02	0.72 ± 0.16*	0.57 ± 0.17*	0.55 ± 0.23*
	CYP3A5	cytochrome P450, family 3, subfamily A, polypeptide 5	0.94 ± 0.14	0.97 ± 0.14	**0.82 ± 0.1**	0.68 ± 0.15*	0.36 ± 0.07*
	AKR1C1	aldo-keto reductase family 1, member C1	1.05 ± 0.11	1.32 ± 0.13*	**1.3 ± 0.22**	**0.95 ± 0.07**	0.62 ± 0.08*
	AKR1C2	aldo-keto reductase family 1, member C2	1.1 ± 0.12	1.36 ± 0.16*	**1.34 ± 0.23**	**0.95 ± 0.08**	0.62 ± 0.1*
	ALDH1A3	aldehyde dehydrogenase 1 family, member A3	0.96 ± 0.08	1.03 ± 0.11	**0.92 ± 0.08**	**0.9 ± 0.05**	0.64 ± 0.17*
Glycolysis/gluconeogenesis	ALDH3A2	aldehyde dehydrogenase 3 family, member A2	1 ± 0.14	1.1 ± 0.19	**0.96 ± 0.16**	**0.84 ± 0.12**	0.71 ± 0.11*
	G6PC	glucose-6-phosphatase. Catalytic subunit	1.29 ± 0.33	0.9 ± 0.3	0.74 ± 0.17*	0.66 ± 0.16*	0.46 ± 0.12*
	ADH1B	alcohol dehydrogenase 1B (class I), beta polypeptide	0.86 ± 0.1	0.82 ± 0.1	0.74 ± 0.11*	0.69 ± 0.13*	0.6 ± 0.11*
	ADH1C	alcohol dehydrogenase 1C (class I), gamma polypeptide	0.76 ± 0.13*	0.65 ± 0.17*	0.5 ± 0.18*	0.39 ± 0.14*	0.17 ± 0.08*
	FBP1	fructose-1.6-bisphosphatase 1	0.97 ± 0.07	0.97 ± 0.1	**0.94 ± 0.04**	**0.9 ± 0.06**	0.74 ± 0.07*
	ADH1A	alcohol dehydrogenase 1A (class I), alpha polypeptide	0.86 ± 0.07	0.87 ± 0.1	0.73 ± 0.15*	0.67 ± 0.16*	0.58 ± 0.17*
	ALDOB	aldolase B, fructose-bisphosphate	0.86 ± 0.19	0.79 ± 0.2	0.67 ± 0.14*	0.37 ± 0.18*	**0.14 ± 0.06**
	ALDH1A3	aldehyde dehydrogenase 1 family, member A3	0.96 ± 0.08	1.03 ± 0.11	**0.92 ± 0.08**	**0.9 ± 0.05**	0.64 ± 0.17*
Fatty acid degradation	ALDH3A2	aldehyde dehydrogenase 3 family, member A2	1 ± 0.14	1.1 ± 0.19	**0.96 ± 0.16**	**0.84 ± 0.12**	0.71 ± 0.11*
	ACSL1	acyl-CoA synthetase long-chain family member 1	1.3 ± 0.1*	1.47 ± 0.16*	1.51 ± 0.15*	1.44 ± 0.11*	**1.2 ± 0.19**
	ACSL5	acyl-CoA synthetase long-chain family member 5	1.04 ± 0.08	1.16 ± 0.06	**1.19 ± 0.22**	**0.97 ± 0.15**	0.76 ± 0.13*
	ADH1C	alcohol dehydrogenase 1C (class I), gamma polypeptide	0.76 ± 0.13*	0.65 ± 0.17*	0.5 ± 0.18*	0.39 ± 0.14*	0.17 ± 0.08*
	ADH1B	alcohol dehydrogenase 1B (class I), beta polypeptide	0.86 ± 0.1	0.82 ± 0.1	0.74 ± 0.11*	0.69 ± 0.13*	0.6 ± 0.11*
	ADH1A	alcohol dehydrogenase 1A (class I), alpha polypeptide	0.86 ± 0.07	0.87 ± 0.1	0.73 ± 0.15*	0.67 ± 0.16*	0.58 ± 0.17*

*Indicates DEGs. **Bold** indicates the exposure time when the pathway was enriched

## Discussion

### The study approach

Elevated levels of free fatty acids are one of the major factors affecting insulin secretion from beta-cells [20]. Measurements of GSIS and intracellular insulin content in the current study demonstrated that up to 1 day’s exposure to palmitate islet beta-cells enhance insulin secretion whereas longer exposure to palmitate leads to a degranulation and to a gradual decline in beta-cell secretory activity. In patients, when the capacity of islets to synthesize and to secrete insulin is diminished to a certain extent, T2DM develops.

In order to protect beta-cells from decline in the function it is important to identify early biological events triggered by the fatty acid exposure and to distinguish whether these pathways are protective/adaptive or deleterious. Such knowledge would provide an opportunity to develop strategies to reverse islet dysfunction and overt T2DM by preventing the negative developments and promoting adaptive processes [21].

To address the issue we identified gene signatures in fatty acid-treated human islets prior to and after the functional decline by combining transcriptomics and bioinformatics approaches. Previously, “omics” approaches helped to identify novel genes and metabolic pathways involved in palmitate-induced beta-cell dysfunction and death. A transcriptomics study on human islets identified 1325 genes differentially expressed after the long-term exposure to palmitate [22]. These DEGs belonged to functional categories “Beta-cell key transcription factors”, “ER stress response”, “Beta-cell signal transduction”, “ATP production”, “Metabolism” etc. [22]. Another transcriptomics study on human islets identified 1860 DEGs which were classified into 14 KEGG pathways including “Metabolic pathways”, “Glycolysis/Gluconeogenesis”, “Fatty acid metabolism” and “Maturity onset diabetes of the young” [23]. Combination of proteomics and lipidomics approaches in our recent study showed that the elevated cholesterol and lipid biosynthesis, altered autocrine insulin signaling and decreased insulin granule maturation may play an important role in palmitate-induced dysfunction of isolated human islets [24]. In our previous study, protein profiling of palmitate-treated INS-1E cells by using 2D gel electrophoresis and MALDI-TOF MS identified 31 differentially expressed proteins with a function in carbohydrate or protein metabolism and Ca^2+^ or mRNA binding [25]. In a similar study, Maris and co-authors identified 83 differentially expressed proteins with a function in ER stress, insulin maturation, intracellular trafficking and generation of harmful metabolites and reactive oxygen species [26].

In the current study, the transcriptomics approach identified 903 DEGs in palmitate-treated isolated human islets. The lower number of DEGs compared with previous studies is due to cut-off that was applied for DEGs: 1.3-fold change after palmitate exposure compared to untreated islets. Of identified genes, 30% were differentially expressed prior to failure of the human islets (up to 1 day of palmitate exposure) and belonged to 8 enriched pathways. These findings underline the importance of understanding early biological changes in islets upon palmitate treatment.

### Analysis of pathways enriched prior to decline of islet function

Four of the eight identified pathways enriched prior to decline of beta-cell function were not enriched in islets with declining function. One pathway of the eight pathways was enriched only when insulin hypersecretion was most prominent. These pathways are discussed in more detail.

The “Mineral absorption” pathway was enriched after 4-h exposure but not at any later time point. The pathway was predicted based on 9 transcripts encoding different subtypes of metallothionein (MT) gene and 1 transcript encoding a solute carrier family 30 (zinc transporter) (Table 1). All the transcripts were elevated in the presence of palmitate. MTs regulate the intracellular level of free zinc, which is known to be essential for the primary functions of beta-cells including insulin biosynthesis and insulin storage. Dysregulation or dysfunction of zinc-transporting proteins leads to impairment of insulin processing and glucose metabolism [27, 28]. Polymorphisms in genes encoding MTs have been related to the development of T2DM [29]. Transgenic mice, with a beta-cell-specific over-expression of MT-2 displayed a significantly reduced beta-cell death and a better preserved insulin production when exposed to streptozotocin [30]. Also, addition of extracellular Zn7-MT-2A potentiated insulin production and secretion from insulin-producing INS-1E beta-cell culture [31]. The abovementioned suggest that an initial increase in MTs transcript level in palmitate-treated human islets is an adaptive mechanism to support insulin synthesis during the insulin hypersecretion.

Another mechanism with a potentially adaptive role is the “Aminoacyl-tRNA biosynthesis” pathway. The pathway was predicted based on 6 tRNA synthetases elevated after 12 h of palmitate exposure (Table 1). Presumably, increased levels of tRNA synthetases enhance insulin biosynthesis, which allows to maintain insulin hypersecretion from palmitate-treated islets. However, due to a short-term up-regulation of these genes, upon longer exposure to palmitate the rate of insulin synthesis falls, causing degranulation of human islets. In obese individuals, hypersecretion of insulin compensates insulin resistance in peripheral tissues caused by elevated levels of circulating fatty acids. Exhaustion of islets would lead to inability to secrete sufficient insulin to compensate insulin resistance and would trigger the development of T2DM.

The “PPAR signalling” and “Adipocytokine signalling” pathways were enriched after 12-h and 1-day exposure to palmitate and included genes with a function in fatty acid and glucose metabolism (Table 1). All the genes from these pathways were elevated. Palmitate-induced upregulation of the genes involved in fatty acid metabolism has been also shown in previous transcriptomics studies [22, 23]. Considering that increased metabolism and beta-oxidation of fatty acids are beneficial for beta cells [32, 33], activation of these pathways is, apparently, an adaptive response to fatty acid exposure. This is supported by previous reports about the protective role of PPAR1 pathway in palmitate-treated beta cells [34]. Although “PPAR signalling” and “Adipocytokine signalling” pathways are not among the enriched pathways after 2- and 7-day exposure to palmitate, most of the genes from these pathways remained at elevated levels (Table 1). It suggests that the contribution of these genes and pathways to beta-cell biology becomes minor and cannot combat the deleterious pathways activated in beta cells upon the long-term exposure to palmitate.

In summary, all the pathways enriched prior to but not during the decline of islet function play an adaptive/protective role in fatty acid-treated human islets.

The “TNF signalling pathway” was predicted based on 8 genes elevated after 1- and 2-day exposure to palmitate (Table 1). Most transcripts in “TNF signalling” pathway encode chemokines. It is known that pancreatic islets produce and secrete a variety of chemokines [35]. In patients, these chemokines may recruit leukocytes into pancreatic tissue and cause beta-cell dysfunction and destruction [35]. However, such inflammatory response is unlikely in our system with isolated islets. Instead, chemokines may interact with numerous chemokine receptors expressed on human islets and, in such way, trigger pathway signalling [36, 37]. The mechanism of chemokine-induced signalling and its effect on insulin secretion is poorly investigated [38]. Considering that activation of “TNF signalling pathway” coincides with insulin hypersecretion one may speculate that this pathway contributes to hypersecretion of insulin rather than exerts direct protective or deleterious effects.

### Analysis of pathways enriched both prior to and during decline of islet function

Three pathways were enriched after 1, 2 and 7 days of palmitate exposure i.e. both prior and during the decline of islet function. The “Fatty acid degradation” and “Glycolysis/gluconeogenesis” pathways were not among the top pathways and were mainly based on the same list of genes as “Metabolism of xenobiotics by P450” pathway (Table 1). Therefore, we will discuss the “Metabolism of xenobiotics by P450” pathway, which was the top pathway after 1 and 2 days and the second top pathway after 7 days of palmitate exposure (Fig. 4).

The “Metabolism of xenobiotics by P450” pathway was predicted based on the reduced expression of genes encoding the detoxification enzymes (Table 1). These enzymes convert drugs and xenobiotics into water-soluble metabolites and play a central role in their detoxification [39]. Elimination of xenobiotics predominantly occurs in liver, kidneys and gastrointestinal tract. However, other tissues/organs are also equipped with diverse detoxification enzymes to minimize the insults caused by xenobiotics [40].

In addition, detoxification enzymes play a role in metabolism of fatty acids via ω-oxidation, a process almost identical to xenobiotic detoxification [41, 42]. During ω-oxidation, which takes place in ER, mixed-function oxidases (CYPs), alcohol dehydrogenases and aldehyde dehydrogenases convert fatty acids into dicarboxylic acid [42]. The formed dicarboxylic acid enters mitochondria or peroxisomes for further metabolism via beta-oxidation [43]. It has been proposed that ω-oxidation is a rescue pathway that allows to eliminate toxic levels of fatty acids that accumulate in the cells when the main β-oxidation pathway is overwhelmed [44]. CYPs that are known to contribute to ω-oxidation is of class 4 [45]. However, it does not rule out that CYP proteins of class 1, 2 and 3, identified in our study, also catalyse ω-oxidation considering that CYPs possess broad substrate specificities [42, 46]. The contribution of glutathione S-transferases and UDP-glucuronosyltransferases to fatty acid metabolism is not known.

Since the detoxification enzymes are important for detoxification of fatty acids, we assume that the inhibition of the “Metabolism of xenobiotics by P450” pathway in human islets upon palmitate exposure is an early deleterious event. It causes accumulation of toxic amounts of fatty acids which contributes to a failure of the islets.

## Conclusion

We propose that in palmitate-treated human islets, at early time points, protective events, including up-regulation of metallothioneins, tRNA synthetases and fatty acid-metabolising proteins, dominate over deleterious events, including inhibition of fatty acid detoxification enzymes, which contributes to potentiation of GSIS. After prolonged exposure of islets to palmitate, the protective events are outweighed by the deleterious events, which contribute to impaired GSIS. The study identifies temporal order between different cellular events, which either promote or protect from beta-cell failure. The sequence of these events should be considered when developing strategies for prevention and treatment of the disease.

## Additional files

Additional file 1: **Table S1.** List of pathways enriched at least at one culture time point. (DOC 63 kb)

Additional file 2: **Table S2.** List of enriched pathways after 4 h of palmitate treatment. (DOC 28 kb)

Additional file 3: **Table S3.** List of enriched pathways after 12 h of palmitate treatment. (DOC 32 kb)

Additional file 4: **Table S4.** List of enriched pathways after 1 day of palmitate treatment. (DOC 37 kb)

Additional file 5: **Table S5.** List of enriched pathways after 2 days of palmitate treatment. (DOC 49 kb)

Additional file 6: **Table S6.** List of enriched pathways after 7 days of palmitate treatment. (DOC 53 kb)

## Abbreviations

BSA: bovine serum albumin
DEGs: differentially expressed genes
DPBS: Dulbecco’s phosphate buffered saline
FFA: free fatty acids
GSIS: glucose-stimulated insulin secretion
HTA: Human Transcriptome Array
T2DM: type 2 diabetes mellitus
