# Supplementary material for: Identification of early biological changes in palmitate-treated isolated human islets
Source: BMC Genomics. 2018 Aug 22;19:629. doi: 10.1186/s12864-018-5008-z (PMC6106933; doi:10.1186/s12864-018-5008-z)
Supplement: Supplementary file 1 — Table S1. List of pathways enriched at least at one culture time point. (DOC 63 kb) [file 12864_2018_5008_MOESM1_ESM.doc]

**Table S1.** List of pathways enriched at least at one culture time point

| **N** | **Pathway name** |
| --- | --- |
|  | Mineral absorption |
|  | Galactose metabolism |
|  | Aminoacyl-tRNA biosynthesis |
|  | PPAR signaling pathway |
|  | Adipocytokine signaling pathway |
|  | Legionellosis |
|  | Fatty acid degradation |
|  | Selenocompound metabolism |
|  | Non-alcoholic fatty liver disease (NAFLD) |
|  | Chemical carcinogenesis |
|  | Metabolism of xenobiotics by cytochrome P450 |
|  | Retinol metabolism |
|  | Drug metabolism - cytochrome P450 |
|  | TNF signaling pathway |
|  | Starch and sucrose metabolism |
|  | Steroid hormone biosynthesis |
|  | Pentose and glucuronate interconversions |
|  | Ascorbate and aldarate metabolism |
|  | Glycolysis / Gluconeogenesis |
|  | Drug metabolism - other enzymes |
|  | Renin-angiotensin system |
|  | Tyrosine metabolism |
|  | Porphyrin and chlorophyll metabolism |
|  | Pancreatic secretion |
|  | ECM-receptor interaction |
|  | Maturity onset diabetes of the young |
|  | Protein digestion and absorption |
|  | Bile secretion |
|  | Rheumatoid arthritis |
|  | Linoleic acid metabolism |
|  | Ovarian steroidogenesis |
|  | Chemokine signaling pathway |
|  | Fat digestion and absorption |
|  | Complement and coagulation cascades |
|  | Leishmaniasis |
|  | Intestinal immune network for IgA production |
|  | Arginine and proline metabolism |
|  | Glutathione metabolism |
|  | Hematopoietic cell lineage |
|  | NOD-like receptor signaling pathway |
|  | Viral myocarditis |
|  | Glycerolipid metabolism |
|  | Staphylococcus aureus infection |
|  | Asthma |
|  | Inflammatory bowel disease (IBD) |
|  | Cell adhesion molecules (CAMs) |
|  | Graft-versus-host disease |
|  | Tuberculosis |
|  | Toxoplasmosis |
|  | Allograft rejection |
|  | Influenza A |
|  | Antigen processing and presentation |
|  | Arachidonic acid metabolism |
|  | Type I diabetes mellitus |
|  | Malaria |
|  | Autoimmune thyroid disease |
