# Supplementary material for: Identification of early biological changes in palmitate-treated isolated human islets
Source: BMC Genomics. 2018 Aug 22;19:629. doi: 10.1186/s12864-018-5008-z (PMC6106933; doi:10.1186/s12864-018-5008-z)
Supplement: Supplementary file 2 — Table S2. List of enriched pathways after 4 h of palmitate treatment. (DOC 28 kb) [file 12864_2018_5008_MOESM2_ESM.doc]

**Table S2.** List of enriched pathways after 4 hours of palmitate treatment

| **q-value** | **Pathway (4h palmitate vs c)** |
| --- | --- |
| 1.47E-13 | Mineral absorption - Homo sapiens (human) |
| 0.061649 | Galactose metabolism - Homo sapiens (human) |
