# Supplementary material for: Identification of early biological changes in palmitate-treated isolated human islets
Source: BMC Genomics. 2018 Aug 22;19:629. doi: 10.1186/s12864-018-5008-z (PMC6106933; doi:10.1186/s12864-018-5008-z)
Supplement: Supplementary file 3 — Table S3. List of enriched pathways after 12 h of palmitate treatment. (DOC 32 kb) [file 12864_2018_5008_MOESM3_ESM.doc]

**Table S3.** List of enriched pathways after 12 hours of palmitate treatment

| **q-value** | **Pathway (12h palmitate vs c)** |
| --- | --- |
| 0.000911 | Aminoacyl-tRNA biosynthesis – Homo sapiens (human) |
| 0.000911 | PPAR signalling pathway – Homo sapiens (human) |
| 0.007667 | Adipocytokine signalling pathway – Homo sapiens (human) |
| 0.022339 | Legionellosis – Homo sapiens (human) |
| 0.030802 | Galactose metabolism – Homo sapiens (human) |
| 0.076938 | Fatty acid degradation – Homo sapiens (human) |
| 0.082389 | Selenocompound metabolism – Homo sapiens (human) |
| 0.082389 | Non-alcoholic fatty liver disease (NAFLD) – Homo sapiens (human) |
