# Supplementary material for: Identification of early biological changes in palmitate-treated isolated human islets
Source: BMC Genomics. 2018 Aug 22;19:629. doi: 10.1186/s12864-018-5008-z (PMC6106933; doi:10.1186/s12864-018-5008-z)
Supplement: Supplementary file 4 — Table S4. List of enriched pathways after 1 day of palmitate treatment. (DOC 37 kb) [file 12864_2018_5008_MOESM4_ESM.doc]

**Table S4.** List of enriched pathways after 1 day of palmitate treatment

| **q-value** | **Pathway (1d palmitate vs c)** |
| --- | --- |
| 3.72E-07 | Chemical carcinogenesis - Homo sapiens (human) |
| 1.28E-06 | Metabolism of xenobiotics by cytochrome P450 - Homo sapiens (human) |
| 4.63E-06 | Retinol metabolism - Homo sapiens (human) |
| 6.28E-05 | Drug metabolism - cytochrome P450 - Homo sapiens (human) |
| 6.28E-05 | PPAR signaling pathway - Homo sapiens (human) |
| 0.000130851 | TNF signaling pathway - Homo sapiens (human) |
| 0.000162113 | Starch and sucrose metabolism - Homo sapiens (human) |
| 0.000174064 | Steroid hormone biosynthesis - Homo sapiens (human) |
| 0.00018389 | Pentose and glucuronate interconversions - Homo sapiens (human) |
| 0.000448162 | Fatty acid degradation - Homo sapiens (human) |
| 0.000798831 | Ascorbate and aldarate metabolism - Homo sapiens (human) |
| 0.002781306 | Glycolysis / Gluconeogenesis - Homo sapiens (human) |
| 0.005453102 | Drug metabolism - other enzymes - Homo sapiens (human) |
| 0.006897313 | Renin-angiotensin system - Homo sapiens (human) |
| 0.014046322 | Galactose metabolism - Homo sapiens (human) |
| 0.019603791 | Tyrosine metabolism - Homo sapiens (human) |
| 0.019603791 | Adipocytokine signaling pathway - Homo sapiens (human) |
| 0.030419957 | Porphyrin and chlorophyll metabolism - Homo sapiens (human) |
