# Supplementary material for: Identification of early biological changes in palmitate-treated isolated human islets
Source: BMC Genomics. 2018 Aug 22;19:629. doi: 10.1186/s12864-018-5008-z (PMC6106933; doi:10.1186/s12864-018-5008-z)
Supplement: Supplementary file 5 — Table S5. List of enriched pathways after 2 days of palmitate treatment. (DOC 49 kb) [file 12864_2018_5008_MOESM5_ESM.doc]

**Table S5.** List of enriched pathways after 2 days of palmitate treatment

| **q-value** | **Pathway (2d palmitate vs c)** |
| --- | --- |
| 2.07E-12 | Chemical carcinogenesis – Homo sapiens (human) |
| 2.62E-11 | Drug metabolism – cytochrome P450 – Homo sapiens (human) |
| 1.05E-09 | Metabolism of xenobiotics by cytochrome P450 – Homo sapiens (human) |
| 2.56E-09 | Retinol metabolism – Homo sapiens (human) |
| 2.31E-06 | Pancreatic secretion – Homo sapiens (human) |
| 2.73E-05 | Steroid hormone biosynthesis – Homo sapiens (human) |
| 0.000458409 | Maturity onset diabetes of the young – Homo sapiens (human) |
| 0.000553241 | Protein digestion and absorption – Homo sapiens (human) |
| 0.000553241 | Drug metabolism – other enzymes – Homo sapiens (human) |
| 0.000786199 | Bile secretion – Homo sapiens (human) |
| 0.001396963 | Starch and sucrose metabolism – Homo sapiens (human) |
| 0.001570132 | TNF signalling pathway – Homo sapiens (human) |
| 0.002315866 | Rheumatoid arthritis – Homo sapiens (human) |
| 0.004384781 | Ascorbate and aldarate metabolism – Homo sapiens (human) |
| 0.005391742 | Linoleic acid metabolism – Homo sapiens (human) |
| 0.005391742 | Ovarian steroidogenesis – Homo sapiens (human) |
| 0.009321478 | Chemokine signalling pathway – Homo sapiens (human) |
| 0.009321478 | Tyrosine metabolism – Homo sapiens (human) |
| 0.009826759 | Pentose and glucuronate interconversions – Homo sapiens (human) |
| 0.014473551 | Glycolysis / Gluconeogenesis – Homo sapiens (human) |
| 0.014473551 | Fat digestion and absorption – Homo sapiens (human) |
| 0.014646171 | Porphyrin and chlorophyll metabolism – Homo sapiens (human) |
| 0.014646171 | Complement and coagulation cascades – Homo sapiens (human) |
| 0.016218667 | Fatty acid degradation – Homo sapiens (human) |
| 0.016218667 | Leishmaniasis – Homo sapiens (human) |
| 0.018013876 | Renin-angiotensin system – Homo sapiens (human) |
| 0.018573397 | Intestinal immune network for IgA production – Homo sapiens (human) |
| 0.020817582 | Arginine and proline metabolism – Homo sapiens (human) |
| 0.02319768 | Glutathione metabolism – Homo sapiens (human) |
| 0.030166769 | Hematopoietic cell lineage – Homo sapiens (human) |
| 0.032142846 | NOD-like receptor signalling pathway – Homo sapiens (human) |
| 0.033085035 | Viral myocarditis – Homo sapiens (human) |
| 0.034045562 | Glycerolipid metabolism – Homo sapiens (human) |
