# Supplementary material for: Identification of early biological changes in palmitate-treated isolated human islets
Source: BMC Genomics. 2018 Aug 22;19:629. doi: 10.1186/s12864-018-5008-z (PMC6106933; doi:10.1186/s12864-018-5008-z)
Supplement: Supplementary file 6 — Table S6. List of enriched pathways after 7 days of palmitate treatment. (DOC 53 kb) [file 12864_2018_5008_MOESM6_ESM.doc]

**Table S6.** List of enriched pathways after 7 days of palmitate treatment

| **q-value** | **Pathway (7d palmitate vs c)** |
| --- | --- |
| 6.19E-10 | Pancreatic secretion - Homo sapiens (human) |
| 4.58E-09 | Chemical carcinogenesis - Homo sapiens (human) |
| 3.77E-08 | Metabolism of xenobiotics by cytochrome P450 - Homo sapiens (human) |
| 5.41E-08 | Drug metabolism - cytochrome P450 - Homo sapiens (human) |
| 2.53E-07 | Steroid hormone biosynthesis - Homo sapiens (human) |
| 5.39E-07 | Protein digestion and absorption - Homo sapiens (human) |
| 7.27E-07 | Staphylococcus aureus infection - Homo sapiens (human) |
| 1.34E-05 | Complement and coagulation cascades - Homo sapiens (human) |
| 3.62E-05 | Retinol metabolism - Homo sapiens (human) |
| 0.00042719 | Toxoplasmosis - Homo sapiens (human) |
| 0.00128489 | Starch and sucrose metabolism - Homo sapiens (human) |
| 0.00128489 | Pentose and glucuronate interconversions - Homo sapiens (human) |
| 0.00146712 | Intestinal immune network for IgA production - Homo sapiens (human) |
| 0.00187604 | Leishmaniasis - Homo sapiens (human) |
| 0.00187604 | Bile secretion - Homo sapiens (human) |
| 0.00196431 | Asthma - Homo sapiens (human) |
| 0.00226998 | Hematopoietic cell lineage - Homo sapiens (human) |
| 0.00294994 | Inflammatory bowel disease (IBD) - Homo sapiens (human) |
| 0.00303115 | Type I diabetes mellitus - Homo sapiens (human) |
| 0.00465809 | Drug metabolism - other enzymes - Homo sapiens (human) |
| 0.00592051 | Ascorbate and aldarate metabolism - Homo sapiens (human) |
| 0.00658043 | Arginine and proline metabolism - Homo sapiens (human) |
| 0.00917408 | Antigen processing and presentation - Homo sapiens (human) |
| 0.00944443 | Graft-versus-host disease - Homo sapiens (human) |
| 0.00944443 | Fat digestion and absorption - Homo sapiens (human) |
| 0.01744687 | Tuberculosis - Homo sapiens (human) |
| 0.01851807 | Glycerolipid metabolism - Homo sapiens (human) |
| 0.01918546 | ECM-receptor interaction - Homo sapiens (human) |
| 0.02344009 | Allograft rejection - Homo sapiens (human) |
| 0.02344009 | Maturity onset diabetes of the young - Homo sapiens (human) |
| 0.02769986 | Ovarian steroidogenesis - Homo sapiens (human) |
| 0.03469335 | Glycolysis / Gluconeogenesis - Homo sapiens (human) |
| 0.03998281 | Porphyrin and chlorophyll metabolism - Homo sapiens (human) |
| 0.04708213 | Small cell lung cancer - Homo sapiens (human) |
| 0.04740034 | Fatty acid degradation - Homo sapiens (human) |
| 0.04863083 | Viral myocarditis - Homo sapiens (human) |
| 0.04948642 | Ether lipid metabolism - Homo sapiens (human) |
| 0.04948642 | Phagosome - Homo sapiens (human) |
| 0.05103194 | Rheumatoid arthritis - Homo sapiens (human) |
